# Spinal maps of motoneuron activity during human locomotion: neuromechanical considerations

**DOI:** 10.3389/fphys.2024.1389436

**Published:** 2024-07-23

**Authors:** Priscilla Avaltroni, Germana Cappellini, Francesca Sylos-Labini, Yury Ivanenko, Francesco Lacquaniti

**Affiliations:** ^1^ Laboratory of Neuromotor Physiology, IRCCS Santa Lucia Foundation, Rome, Italy; ^2^ Department of Systems Medicine and Center of Space Biomedicine, University of Rome Tor Vergata, Rome, Italy

**Keywords:** locomotion, spinal cord, motor pools, muscle innervation, spinal cord imagery, neurorehabilitation, interactive locomotion

## Abstract

The spatial segmental location of motoneurons in the human spinal cord is influenced by both evolutionary and functional principles tending to optimize motor control, reflex integration, and adaptation to the demands of movement. Bearing in mind the biomechanics of limb muscles, it is logical to examine how motoneuron activity clusters functionally during typical daily activities like walking. This article provides a summary of advancements in the study of spinal maps of motoneuron activation during human locomotion by reviewing data gathered over ∼20 years. The effects of child development, aging, and neurological disorders show the salient characteristics of spinal segmental activity during different human locomotor tasks and conditions. By exploiting the neuromechanics of the spinal motor circuits, that is, the link between motoneuron activity and gait mechanics, neuroprosthetics and other focused treatments may better help individuals with locomotor impairments.

## Introduction

The central nervous system orchestrates intricate patterns of muscle activation through motoneurons (MNs) constituting the “final common pathway” (Sherrington, 1906). The organization of the evolutionary ancient spinal cord motor apparatus is tightly related to the executive function of the spinal circuitry, which is close to the periphery. Since locomotion is a typical activity in daily life, it makes sense to consider the anatomical and functional clustering of motoneurons in the context of biomechanical features of limb muscles and underlying neuromechanics of locomotor movements. Human locomotion is a remarkable feat, characterized by the seamless coordination of numerous muscles and joints to accomplish movement with precision and efficiency. Understanding the neural mechanisms that underlie this intricate control is a fundamental pursuit in neuroscience, biomechanics, and rehabilitation sciences. Traditionally, neuroimaging and investigations into motor control focused on the cortical regions of the brain, with an emphasis on the primary motor cortex and descending pathways to the spinal cord. However, recent advances in neuroimaging and neurophysiological techniques have opened up new avenues to explore the neural circuitry at the spinal level, providing insights into the recruitment and organization of motoneurons during locomotion. This approach is of particular interest for modulating neuronal circuits enacting locomotion, because the executive component of the locomotor pattern generation circuitry and the common final pathway, that is the motoneurons, are located in the spinal cord (Grillner and El Manira, 2015). Moreover, as we will argue in a later section, whether or not motoneurons are an integral part of the Central Pattern Generators (CPGs) of locomotion, they most likely contribute to shaping the rhythm and patterns specified by CPGs. To date, non-invasive electrophysiological recordings have made significant contributions to the temporal and spatial data on motoneuron recruitment during normal human locomotion. These data provided insights into the evolutionary adopted neuromechanics of motor pool activity in the human spinal cord and the adaptive plasticity of spinal maps under various conditions.

In this review, we consider the main advancements in the study of spinal maps of motoneuron activation during human locomotion, drawing on data collected over about 20 years and concentrating on the functional properties of motor pool activity in both health and disease. The topographical (anatomical) arrangement of the motor pools in the human spinal cord will be examined first, followed by a complementing comparative study in animals. After that, we shall discuss the neuromechanics and experimental evaluation of motor pool activity in various human gaits, as well as how it reorganizes with age as infants mature and learn to move around. Finally, we explore the application of spinal maps to monitoring and stimulation of the spinal locomotor circuitry to assist patients with locomotor deficits in returning to their normal movement patterns.

## Muscles innervation and motor pool arrangements in the lumbosacral spinal cord

We start with brief historical notes on seminal work on the anatomical and connection structure of the spinal motor system. Although the problem of muscle innervation had been previously investigated by several authors, the first rigorous study of the muscle innervation by the lumbo-sacral plexus was made by Sherrington (1906) in an extraordinary, scholarly paper of 152 pages plus 12 Plates, comparing frog, rat, rabbit, cat, dog, and monkey (Macacus). The anatomical localization of motor neurons in the ventral spinal cord was thoroughly studied by George Romanes, who first described the cephalo-caudal development of cell columns in rabbit embryos (Romanes, 1941), and then described the multilayered topographic columnar system that links the spatial arrangement and biomechanical features of limb muscles to the settling location and clustering of motor cells in the spinal cord of the cat (Romanes, 1951). A little later Sharrard arrived at the same general plan by using comparable procedures to the lumbosacral region of the human spinal cord (Sharrard, 1955). These early pioneering works laid the foundation for subsequent, more recent research and insights into the molecular programs that determine the position of the motor pools and how the position of motor neurons shapes circuit assembly (Jessell et al., 2011). They also shed light on the ways that neuronal placement affects sensory-motor connections and may be relevant to the structure of circuits in other parts of the central nervous system.

**FIGURE 1:**
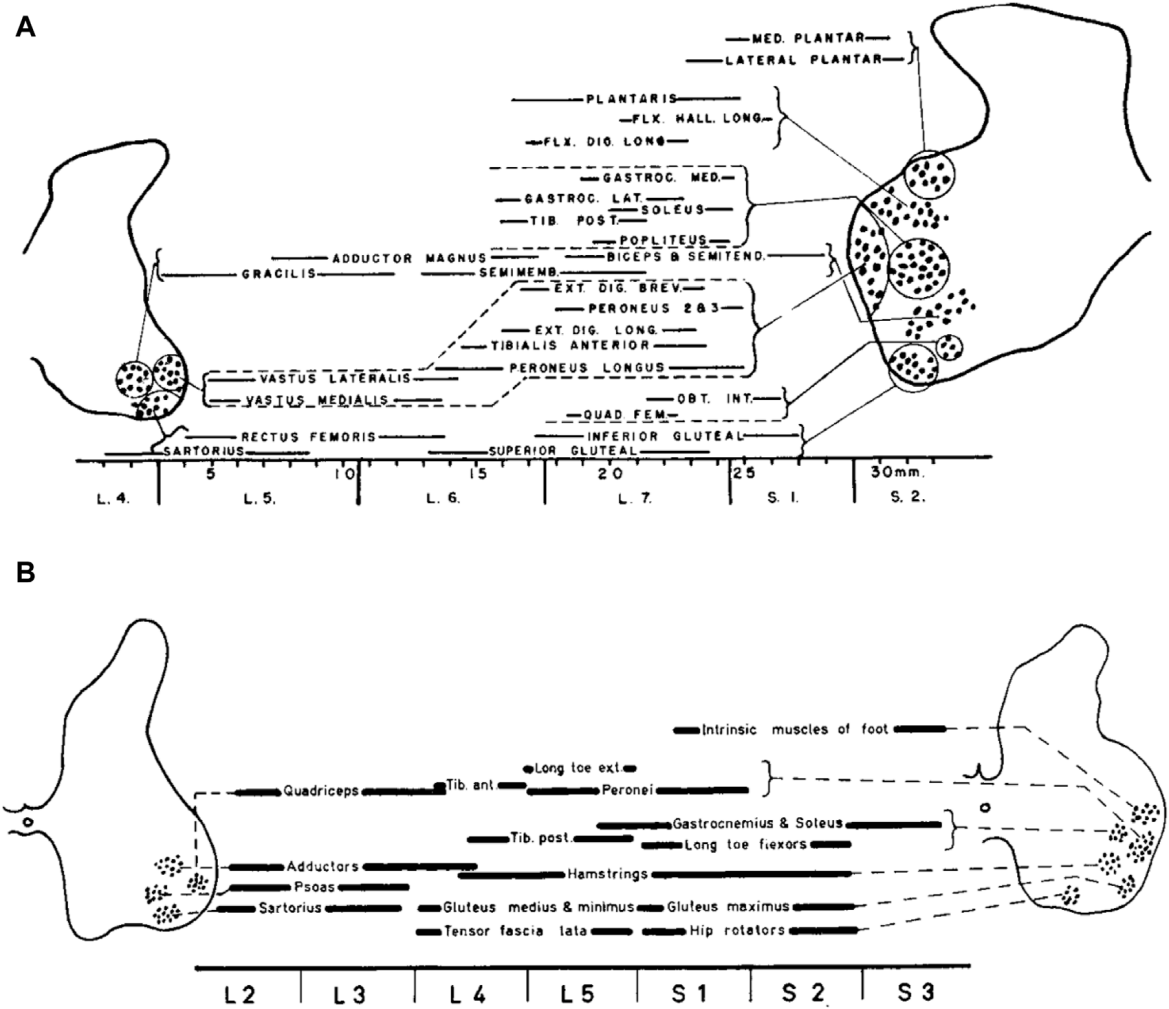
The organization of motor pools in the lumbosacral spinal cord of the cat **(A)** and human **(B)**. Diagrams show the position of the motor cell groups and the muscles they supply (reproduced from Romanes, 1964, with permission from Elsevier). The cat’s motor pool layout is derived from Romanes (1951), while the human data are constructed from Sharrard (1955).

Figure 1 illustrates the organization of motor pools in the lumbosacral spinal cord of the cat (top) and human (bottom), showing the position of the motor cell groups and the muscles they supply. Motoneurons in the spinal cord innervating limb muscles are clustered into spatial pools that occupy well defined locations in the anterior horns. The set of motor pools that innervates muscles exerting synergistic functions are grouped together, forming mini-columns or columels, positioned along the rostrocaudal axis of the lumbar spinal cord. Motor columels exhibit a positional plan; in general, cell columns lying ventrally in the anterior horn supply the muscles in the proximal part of the lower limb, whereas those lying dorsally supply the more distal muscles (Romanes, 1951). Even though these and other animal studies have been helpful in offering a parallel for the exploration of human limb innervation, they cannot provide the whole answer for humans. In contrast to animal studies, experimental studies of the innervation of muscles segments in the human lower limbs were largely limited, until Sharrard’s research. The findings derived from the investigations about the stimulation of motor nerve roots and motor root lesions made possible the reconstruction of the innervation of the lower limb muscles in humans (Sharrard, 1964). These results agree (except for minor differences) with the findings derived from an investigation about the distribution of paresis and paralysis in the human lower limb muscles, as resulting from the loss of motoneurons in the lumbo-sacral spinal cord both in children (Sharrard, 1955) and adults (Routal and Pal, 1999; Kendall et al., 2005).

Instead of being dispersed randomly throughout the ventral horn, the spinal motoneurons responsible for innervating a single limb muscle are grouped into spatially coherent “pools”. In humans, the central L3, L4, L5, and S1 segments of the lumbosacral enlargement have more motor cells overall (Tomlinson and Irving, 1977). In contrast, the boundary segments L1 and S3 have 10–20 times fewer MNs overall than L3, L4, L5, and S1 segments, and the L2 and S2 segments have two–three times fewer MNs overall than those segments. The slight variations in the relative locations of MNs between humans and cats (Figure 1) can be attributed in part to differences in the shape of the ventral horns between the two species. Additionally, Sharrard notes more distinct groups in humans than those seen in cats, which could possibly point to a higher level of cell group separation in humans. There is additional evidence from more recent human research that the cell groups in the lumbosacral region are more distinct than those in the cervical region. Thus, neuronal groups were fewer, larger, and, on many levels, better defined in the lumbosacral region than in the cervical region, where they were more numerous but smaller and less distinct (Routal and Pal, 1999). Nevertheless, overall, both human and cat data show a similar general topographic plan of muscle innervation. An essential feature of anatomical maps of MN location is their muscle-specific location along the rostrocadual, mediolateral, and dorsoventral axes of the lumbar spinal cord. In general, each muscle is innervated by several spinal segments, and each segment supplies several muscles (Sharrard, 1964; Kendall et al., 2005).

The vast majority of neurons in the spinal cord are involved in sensory-motor processing, interneurons that facilitate communication between different levels of the spinal cord and supraspinal centres, and various other functions essential for coordinating movement, reflexes, and autonomic functions. Motoneurons represent a relatively small proportion of the overall neuronal population in the lumbosacral spinal cord. However, they play a critical role as the final common pathway for motor control. For locomotion, they may have additional roles, as we will argue later. It is also worth stressing that, in the developing spinal cord, motoneurons are the first active neurons, and form local patterned ensembles with neighbouring neurons (Hanson and Landmesser, 2003; Warp et al., 2012; Wan et al., 2019). Moreover, motoneuron positioning is tightly related to sensory-motor connectivity and the assembly of spinal motor circuits (Jessell et al., 2011; Arber, 2012; Kiehn, 2016). The reconstruction of the innervation of human lower limb muscles (Figure 1B) and registration of muscle activity during locomotion and other tasks have allowed many researchers to investigate the spatiotemporal activity of the spinal motor pools, as measured by spinal topography. These findings will be discussed in more detail in the following sections.

## Spinal maps assessed by mapping muscle activity onto the rostrocaudal location of MNs

**FIGURE 2:**
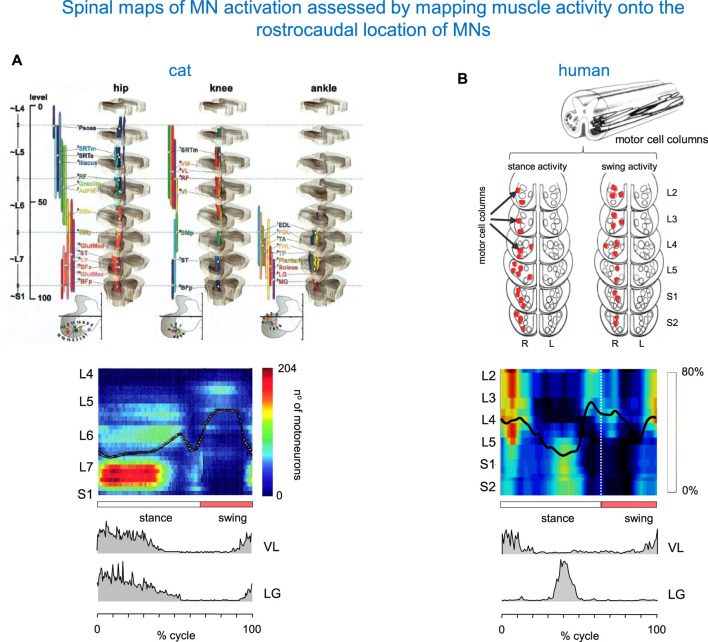
Spinal maps of MN activation during locomotion assessed by mapping muscle activity onto the rostrocaudal location of MNs. **(A)** Spatiotemporal map in the cat (adapted from Yakovenko et al., 2002 with permission). On the top: anatomical model of the organization of the MN pools. On the bottom: intramuscular EMG activity of vastus lateralis (VL) and lateral gastrocnemius (LG) muscles (redrawn from Smith et al., 1998). **(B)** A similar diagram in the human spinal cord (modified from Ivanenko et al., 2006). The upper panel: a sketch of motor cells for S2–L2 segments (adapted from Sharrard, 1955; Sharrard, 1964). Columns that display their major activity during the stance and swing phases of the right (R) leg are marked in red. The centre of activity in the spinal maps is shown by the black lines. Pattern is plotted vs. normalized gait cycle.

The method for assessing the spatiotemporal maps of alpha-MNs activation during locomotion was first developed for cats (Yakovenko et al., 2002), and the technique was then applied to humans (Grasso et al., 2004; Ivanenko et al., 2006). It consists of simultaneous kinematic and electromyographic (EMG) recordings in several muscles (that provide an indirect measure of the net firing of MNs of those muscle in the spinal cord at any particular moment), and mapping the recorded patterns of muscle activity onto the approximate rostrocaudal location of the MN pools within the spinal grey matter in the spinal cord. Yakovenko et al. (2002) constructed the maps from anatomical data on MN localization obtained by Vanderhorst and Holstege (1997) and from records of EMG activity of 27 hindlimb muscles during locomotion of intact cats to characterize the spatiotemporal migration of motor pool activity in the cat spinal cord during normal locomotion (Figure 2A). A rostrocaudal oscillation of activity in hindlimb MN pools emerged from these maps (see centre-of-activity, Figure 2A). During the stance phase of locomotion, there was significant motor neuron activity in the caudal part of the lumbosacral enlargement. At the stance-to-swing transition, a transient focus of activity developed in the most caudal region, and during the swing phase the activation’s focal point moved to the rostral region of the lumbosacral enlargement. The developed model of the spinal cord generated from these digitized data sets (Figure 2A) proved to be a valuable, non-invasive technique for similar applications in humans.

The utilization of non-invasive neuroimaging modalities, such as functional magnetic resonance imaging (fMRI), has significantly advanced our ability to visualize and map the neuronal activity of the human spinal cord and its impairment (Lawrence et al., 2008; Powers et al., 2018; Landelle et al., 2021; Hemmerling et al., 2023; Kaptan et al., 2023; Stroman et al., 2014). The recent advancements in fMRI sequences that can simultaneously target the brain and spinal cord have opened up new avenues for studying the neural mechanisms at multiple levels of the CNS (Vahdat et al., 2015; Kinany et al., 2023). fMRI of the lumbosacral spinal cord can also be used to detect neuronal activity during some lower limb movement tasks (Kornelsen and Stroman, 2004; Kornelsen and Stroman, 2007). However, comparable functional imaging of motor pool activity in the human spinal cord remains difficult to apply to walking. Apart from technical challenges like movement artefacts, *etc.*, it is unclear which neural structures in the spinal cord can be evaluated by functional magnetic resonance imaging, and it is challenging to discern between MN activations and interneuron activations related also to the sensory inputs that always accompany movement. Therefore, the approach developed for cats (Yakovenko et al., 2002) represents a viable and perhaps unique alternative for documenting the spatiotemporal maps of alpha-MNs during human locomotor tasks. Despite limitations in the experimental assessment of motor pool activity, the study of spinal maps of motoneuron activation represents a promising avenue of research, bridging the gap between the brain and the spinal cord in the context of the control of human locomotion in both health and disease (Scivoletto et al., 2007).

By employing this technique, Figure 2B shows a diagram of spinal MN activation during human walking. The lumbosacral map of MN activation obtained in healthy humans using this method has some of the same features as that of Yakovenko et al. (2002). In both sets of maps (Figure 2), the locus of maximum concentration of MN activity (computed as Centre of Activity, CoA) oscillates rostro-caudally in the spinal cord during swing and stance, and spinal activity jumps from one region to the other. However, there are some differences between the two species. In humans, the first activation component is centred near the origin (time of heel strike) at ∼10% of the cycle. It appears primarily in the mid and upper lumbar segments (L4–L2) and extends into the lower thoracic segments. The next major activation phases are centred at ∼45% (activity in the sacral segments S1, S2, while activity in the lumbar segments is much reduced), ∼60% (activity in mid and upper lumbar segments occurring around lift-off), and 95% of the cycle (focused on all spinal segments) (Figure 2B). In our previous studies, we evaluated the sensitivity of the spinal locomotor output to inter-stride and inter-individual differences, the number of recorded muscles and potential crosstalk (Ivanenko et al., 2006; 2013; Cappellini et al., 2010).

Despite individual variations in the segmental level of spinal activation, the major features depicted in the stride-averaged maps are representative of the general trends in individual strides (La Scaleia et al., 2014a). Also, despite some minor differences apparent in the colour-scale maps, the segmental motor pool output estimated from the activity of 18 or 12 ipsilateral muscles is roughly similar, and captures four major loci of activity in the gait cycle (Ivanenko et al., 2013) illustrated in Figure 2B. On the one hand, this result shows that the method is robust, and can be applied even with a lower number of recorded muscle activity, as would be the case in some pathological cases. On the other hand, the result indicates that the method does not provide a sensitive measurement of the activation of motoneuron pools of different muscles. For the latter, one would need an extension of high-density muscle fibre electromyograms (Sartori et al., 2017) to multiple muscles recording during locomotion, which is currently not feasible.

If we now return to the comparison with spinal maps in cats, we notice that in the latter species, lower lumbosacral spinal segments contribute earlier in stance, whereas L4 and L5 activity occurs later in stance (Figure 2A). This is also seen in the activity of lateral gastrocnemius (LG). In humans, an activation peak of LG occurs late in stance at the lower lumbosacral region. In cats, however, it occurs at the start of stance. The difference in the timing of activity of the proximal extensors (such as vastus lateralis VL, Figure 2) likely reflects specific features of locomotion in each animal species, including the biomechanics of quadrupedal (knee-flexed) *versus* bipedal (leg extended) gait.

## “Neuromechanics” of motor pool activity in human gaits

In line of principle, the spatial distribution of motoneurons might play an important role in embedding musculoskeletal dynamics and the total assembly of spinal motor circuits. In the cervical enlargement of macaques, the distances between motoneuron pools innervating synergistic muscles are the shortest, followed by those innervating antagonistic muscles (Jenny and Inukai, 1983; Taitano et al., 2024). This kind of spatial arrangement is compatible with models of locomotor central pattern generators (CPG) (Grillner and El Manira, 2015; Ausborn et al., 2018) involving the co-activation of synergistic muscles and the reciprocal inhibition of antagonistic muscles (Taitano et al., 2024). An essential aspect of segmental grouping of MNs of different muscles is their rostrocaudal distribution. The rhythmogenic capacity of the CPG is distributed along the lumbar cord, with a rostrocaudal excitability gradient (Lev-Tov et al., 2000; Kiehn, 2006). The capacity for segmental rhythmogenesis and the rostrocaudal propagation of spinal cord activity is possibly conserved in the course of evolution (Falgairolle et al., 2006). It is also important to note a similar relative rostrocaudal organization of both anatomical and functional (e.g., assessed by microstimulation) spinal maps of the lumbosacral motoneuronal pools (Toossi et al., 2019). We will consider here the latter aspect (functional spinal maps of MN activity) in the context of human bipedal locomotion.

It is important to remark that the spatiotemporal maps of activity provide only a global view of ensemble alpha-motoneurons activity, and give indirect information about the activity of premotor interneurons, which represent the core of CPGs, at least in animal models (Kiehn, 2016; Grillner and El Manira, 2020). Nevertheless, the issue of whether or not motoneurons are directly part of CPGs remains unsettled (Marder, 1991; O’Donovan et al., 1998; Mentis et al., 2005). Indeed, while motoneurons are often considered only the last relay from the central nervous system to the muscles, there is evidence in several animal species that they can play a role in shaping the locomotor rhythm and patterns by providing feedback to upstream circuitry (Barkan and Zornik, 2019). Thus, in zebrafish, motoneurons are electrically coupled via gap junctions to excitatory V2a interneurons in the locomotor CPG (Song et al., 2016). In rodents, motoneurons can modulate locomotor CPG activity by projecting onto Renshaw cells, other motor neurons (Bhumbra and Beato, 2018), and V3 interneurons (Chopek et al., 2018). It is also worth noting that, in mice, brainstem commands to initiate rhythmic locomotor activity are tightly channelled to motoneurons via modular pathways involving an entry point in the spinal cord, an immediate executor module, and a premotor module (Hsu et al., 2023). This then shows how motoneurons are an integral part of the chain of commands leading to locomotor rhythm and pattern generation.

As we noted above, the human spinal maps of activation (Figure 2B) have some specific features related to the idiosyncratic gait style involving erect bipedalism. Humans have many musculoskeletal specializations for bipedalism (Bramble and Lieberman, 2004), with walking and running being the most common forms of human locomotion. They show highly automated rhythmic movements with a relatively stereotyped pattern of muscle activation, which most likely reflects both the activity of underlying pattern generators and proprioceptive feedback. In bipedal humans, keeping the leg fully extended during midstance helps to reduce joint torques, muscle forces and energy expenditure during limb loading (Alexander, 1991). This, in turn, calls for a prominent lumbar activity at midstance (related to the activity of proximal leg extensors), and a functional separation of the lumbar and sacral segmental activities (Figure 2).

An essential feature of some schemes of spinal activation is the pulsatile nature of the major part of the motor output (Cappellini et al., 2006), consistent with “drive pulse” rhythmic elements in the spinal circuitry of animals (Giszter et al., 2007). The concept of drive pulses is germane to the original idea of unit burst generators put forth by Grillner (1981), Grillner and El Manira (2020). While the exact mechanisms underlying burst generation in mammals are still incompletely understood, the picture in an old vertebrate, the lamprey, has been described in detail (Grillner and Kozlov, 2021). The segmental lamprey network involves a pool of interacting excitatory interneurons (Buchanan and Grillner, 1987). Burst initiation is triggered when excitatory interneurons receive excitatory descending drive. Burst termination depends on an initial increase of the level of intracellular Ca2+ activating calcium-dependent potassium channels, which gradually hyperpolarize the excitatory interneurons.

The idea of the spinal circuitry with constraints on the temporal functional connectivity of hypothetical pulsatile burst generators is supported by the factorial analysis of multi-muscle EMG activity patterns (Ivanenko et al., 2004) and by biomechanical modelling studies (Neptune et al., 2009). It is noteworthy that the main loci of MN activity during forward locomotion are associated with specific spinal segments and with specific biomechanical actions (Lacquaniti et al., 2012).

**FIGURE 3:**
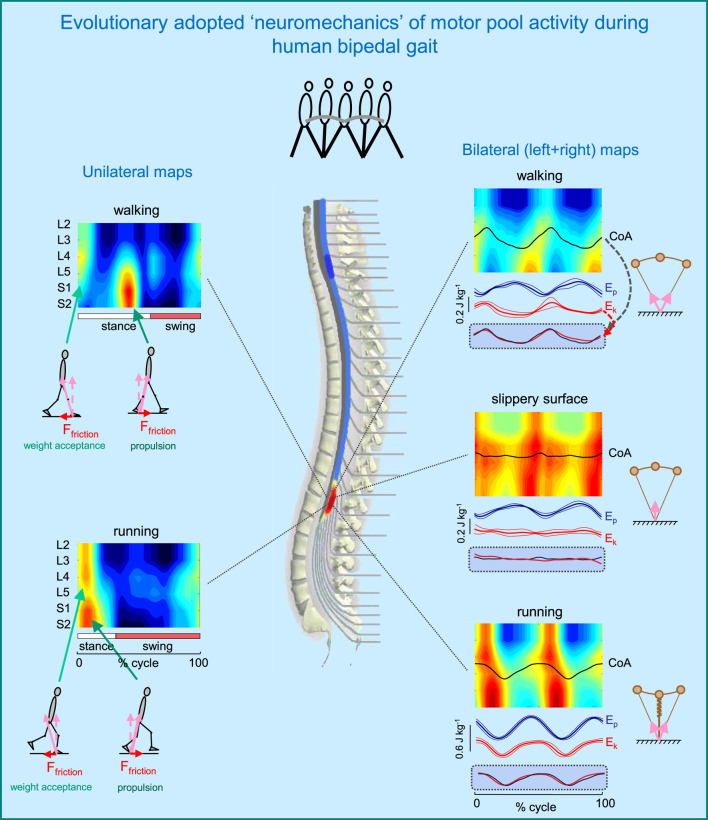
Evolutionary adopted MN grouping, motor pool activity, and motor wave propagation in the human spinal cord (adapted from Cappellini et al., 2010). On the left–unilateral maps of MN activity, on the right–bilateral maps (when patterns of each half cord activation were collapsed together to obtain the total bilateral motor output). Spinal maps are illustrated for different human gaits (normal walking, walking on a slippery surface, and running). Arrows schematically indicate the resultant ground reaction forces during weight acceptance and propulsion phases (with corresponding horizontal and vertical components). Potential (E_p_) and kinetic (E_k_) energies (±SD) of the centre-of-mass (COM) are also shown on the right. The black curves correspond to the centre of MN activity (CoA), which is superimposed on the COM kinetic energy curves for each bilateral map on the bottom. Note systematic oscillations of CoA from S1 to L4 segments (twice per stride) during walking and running and high correspondence of these CoA oscillations to kinetic energy. In the absence of friction (slippery surface) there is a lack of both motor wave propagation (CoA) and changes in E_k_.

Figure 3 illustrates such idiosyncratic motor pool activity during human walking and running. There are two types of activity maps displayed: the ipsilateral (left panels) and bilateral (right panels) maps. The ipsilateral maps of the lumbosacral enlargement, provide an indirect reflection of the primary spatiotemporal torque patterns that the corresponding limb exerts. The two major loci of activity, around touchdown and end of stance, are associated mostly with the activity of proximal (quadriceps) and distal (ankle plantar-flexors) extensors during weight acceptance and propulsion, respectively (Figure 3, left panels). In addition, lateral trunk stabilization may also be involved during weight acceptance, including the activation of tensor fascia latae and gluteus medius, which are also innervated from the lumbar segments. It is also worth noting that extensor limb muscles have a significantly larger physiological cross-sectional area than flexor muscles (Ward et al., 2009). During walking, the two prominent loci of activation occur at ∼5 and 45% of the gait cycle. During running, they are closer to each other, in parallel with the shorter stance duration (Figure 3, left lower panel).

Although the activation patterns may be localized at different spinal segments in a gait-specific manner, the pulsatile nature of motor pool activity is conserved during other human gaits, such as skipping, backward walking, and walking on inclined surfaces (Ivanenko et al., 2008). During skipping, a gait mode frequently displayed by young children (as well as by astronauts locomoting on the Moon) and having features of both walking (double support phase) and running (flight phase), the timing of the major peaks of sacral activation corresponds perfectly to those during both walking and running (Ivanenko et al., 2008). Since both sacral temporal bursts are present (superimposed) during skipping, this result suggests that walking, running and skipping may share the same timing generator circuitry. During backward walking, the propulsion burst at the end of stance is linked to the lumbar rather than sacral segments as in forward walking, engaging quadriceps muscle instead of triceps surae (Ivanenko et al., 2008), while during walking on inclined surface the relative intensity of lumbar and sacral loci of activity at the onset and end of stance depends on the slope (Dewolf et al., 2019). While lumbar motor pools get more engaged when both positive and negative slopes increase, sacral motor pools become less active at negative slopes and more active at positive slopes when compared to level walking. These findings are consistent with ‘drive pulse’ rhythmic elements in the spinal circuitry, though with a differential involvement of the lumbar and the sacral motor pools depending on the slope and walking direction, which should likely be taken into account when using spinal neuroprosthetics for rehabilitation of adaptive gait (see the section “*Spinal maps for evaluating and neuromodulating disease-related locomotor output*”).

The neural substrates that control elasmobranch fins and mammalian limbs, including a rostrocaudal propagation of motor waves in the spinal cord, had already evolved millions of years ago (Falgairolle et al., 2006; Grillner, 2018). The layout of the motoneurons in the human spinal cord shows a specific topography (Sharrard, 1964), and this anatomical arrangement might underlie a way in which leg muscles work when moving the centre-of-mass (COM) during terrestrial locomotion. However, this question has not been specifically addressed in prior research, except for a few studies on a rostrocaudal propagation of motor waves in trunk muscles (e.g., de Sèze et al., 2008). In our previous study (Cappellini et al., 2010), we looked at the bilateral maps of MN activity to address this question. The rationale behind a bilateral analysis is that coordinated action of all the muscles in both legs, rather than the muscles of only one leg, determines the motion of the body COM. The bilateral maps were obtained by collapsing together the patterns of each half cord activation to characterize the total bilateral motor output. These maps for different types of human gait are illustrated in Figure 3 (right panels). During walking and running, the centre of bilateral motoneuron activity (CoA) exhibits two major rostrocaudal activation oscillations associated with the left and right heel strike and shear (friction) forces during step-to-step transitions. Gravitational potential energy E_p_ and forward kinetic energy E_k_ of the COM show out-of-phase and in-phase behaviour during human walking and running, respectively. These behaviours are consistent with an approximation of walking as vaulting gait (inverted pendulum) and running as a bouncing gait (leg spring-loaded during stance) (Figure 3, upper and lower right panels). Interestingly, the rostrocaudal displacements of the CoA of bilateral motoneurons mirror the changes in the kinetic energy of the COM motion during both walking and running. Moreover, when we examined walking on a slippery surface, which considerably reduces shear forces at step-to-step transitions, both the oscillations of the COM kinetic energy and the CoA caudal and rostral shifts were significantly smaller than those for normal walking (Figure 3, right middle panel). A striking correspondence of the rostrocaudal propagation of spinal cord activity with changes in the energy of the COM motion suggests a possible neural correlate of the biomechanics of bipedal gait related to the underlying principles of the evolutionary adopted motoneuron grouping. This idea is tightly related to Taga’s model (Taga, 1995) predicting walking movements from the dynamic entrainment among the neural and musculo-skeletal systems and the environment (see also Lacquaniti et al., 1999).

## Spinal maps in locomotor development

Neuroimaging of the spinal cord in animals has become a potent technique for determining the neurogenesis and evolutionary origins of the spinal locomotor networks. For instance, neuroimaging of the developing spinal cord may reveal the emergence of coordinated activity at single-cell resolution. Cellular-resolution analysis of population activity in zebrafish demonstrated that motoneurons form first pattern ensembles followed by global synchronization based on threshold network size and then by commissural interneurons that establish left-right coordination (Warp et al., 2012; Wan et al., 2019). Machado et al. (2015) used two-photon imaging coupled with spike inference to measure locomotor firing in hundreds of motor neurons in isolated mouse spinal cords. Interestingly, their findings highlighted the evolutionary primacy of flexor pattern generation, since after reversion of motor neuron identity in mutant mice, almost all firing patterns became distinctly flexor-like. Pham et al. (2020) recorded two stepping bouts and discovered a significant amount of redundancy among spinal locomotor circuits using neuroimaging, which involves labelling spinal neurons and recording population activity in the adult mouse spinal cord. Overall, neuroimaging studies in animals show that motor neuron identity directs locomotor circuit wiring and that the coordinated activity is gradually emerging in the developing spinal cord. The emergence of locomotor activity and systematic age-related changes in the spinal activity maps can also be studied in humans using the non-invasive method originally developed by Yakovenko et al. (2002) (Figure 2).

**FIGURE 4:**
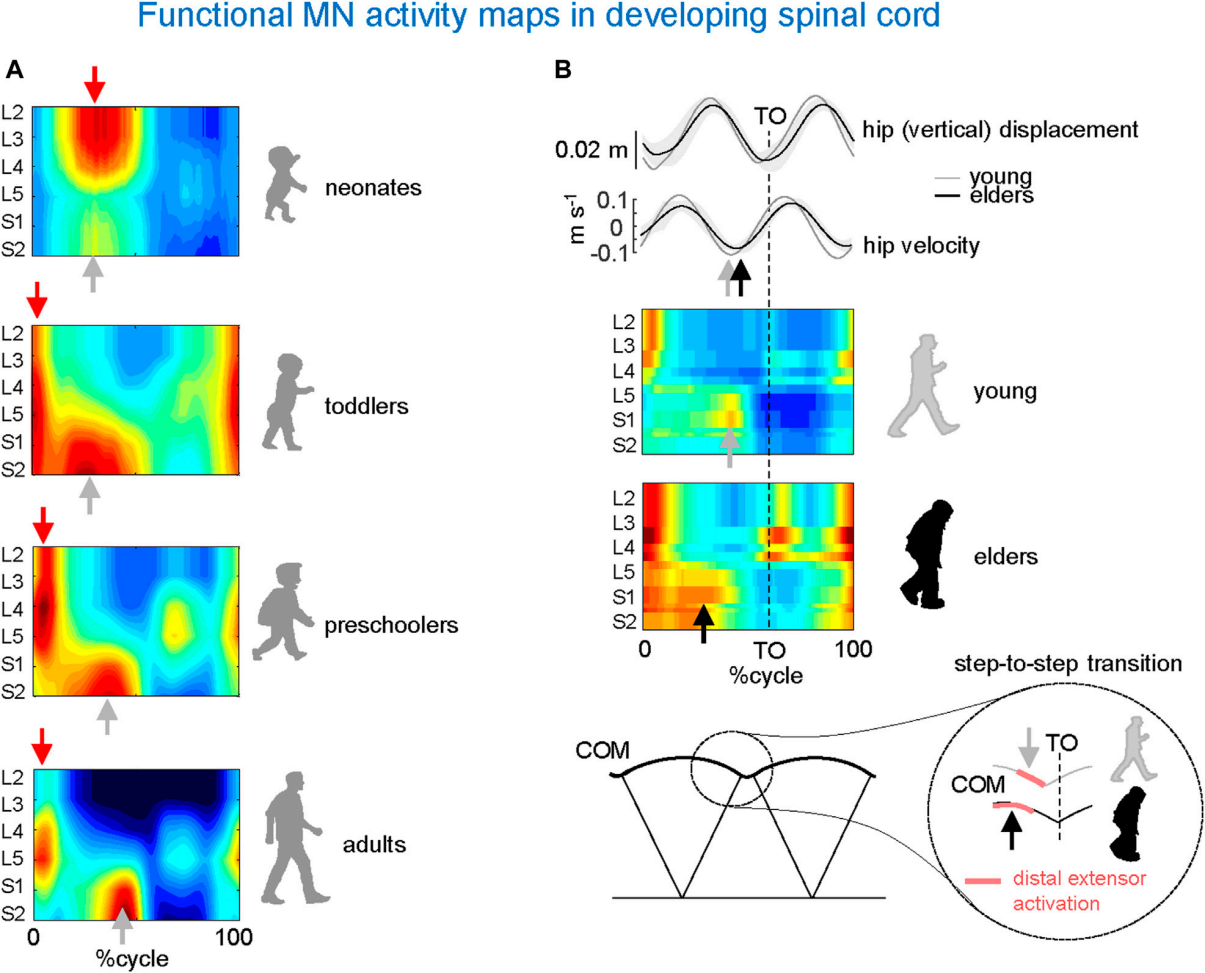
Age-related changes in MN activity maps in developing spinal cord. **(A)** Unilateral spinal maps in neonates, toddlers, preschoolers, and adults (adapted from Ivanenko et al., 2013). Note a gradual functional reorganization of motor pool activity from a quasi-simultaneous activation of lumbar and sacral segments in neonates to a clearly dissociated activity in adults. This aligns once more (as seen in Figure 3) with the close connection between the biomechanics of COM motion (neonates’ lack of propulsive step-to-step transition activity) and motor wave propagation in the spinal cord. **(B)** Impact of aging related to the loss of anticipated COM step-to-step transition strategy in elderly: due to the lack of late push-off prior to toe-off (TO) from the trailing leg (sacral activity shifts toward an earlier midstance), the down-to-up redirection of the COM velocity starts later in older than in young adults (adapted from Dewolf et al., 2021).

Figure 4A provides an example of the results obtained from these investigations conducted on human participants (Ivanenko et al., 2013). To this end, the spinal segmental output was examined during stepping in newborns, toddlers, preschoolers, and adults. A remarkable feature of newborn stepping is a rostrocaudal coactivation of motoneurons, which are activated mostly during the stance phase of the cycle. A higher overall activation of lumbar *versus* sacral segments (Figure 4A, upper panel), may reflect a gradient in rostrocaudal excitability in line with the animal studies on a greater rhythmogenic capacity of rostral (D7–D10 in turtles, L1–L3 in rodents, L3–L5 in cats) vs. caudal segments (Cazalets and Bertrand, 2000; Lev-Tov et al., 2000; Vinay et al., 2002; Kiehn, 2006). A second burst of activity during neonatal stepping is observed during swing related to the activation of ankle dorsiflexors. Notice that flexor activity during swing in neonates is probably underestimated due to the difficulty of recording from the main hip flexor, iliopsoas.

In adult humans, the upper lumbar pattern generator activity may also function as a major pacemaker (Gerasimenko et al., 2010), whereas the sacral generator could play a subordinate role for adaptation to specific foot-support interactions (Selionov et al., 2009). Further insights into the developmental process can be gained by analysing the segmental structure of motoneuron activity in older children. In toddlers, motor pool activity shifts to the sacral cord segments at midstance, while lumbar and sacral motoneurons are activated around touchdown. Preschoolers show a roughly similar pattern, but with a greater separation between the activity at the beginning of stance and the rest of the stance pattern (Figure 4A). The separation becomes more prominent with further development with progressively briefer motoneuron activations in adults, resulting in distinct bursts of activity in the spinal maps (Figure 4A, lower panel). Thus, the findings suggest a developmental sequence that begins with a common patterning of motor pool activity in the entire lumbosacral cord, which later progresses with a separate patterning of activity in the lumbar and sacral spinal segments consistent with separate maturation of lumbar and sacral pattern generators. In sum, development of human locomotion from the neonate to the adult starts from an overall rostrocaudal excitability gradient, and entails a progressive functional remodelling of the pattern generation circuitry.

## Spinal maps in the elderly

The process of remodelling and organization of the segmental structure of motoneuronal activity may restart with ageing. Changes in gait features of locomotion occur as a result of increased risk of falling (Maki, 1997) and/or health status in older adults (Studenski et al., 2011). Factors associated with age-related changes include decreased grey matter volume (Good et al., 2001), the number of motor cortical (Henderson et al., 1980) and spinal motor neurons (Doherty, 2003), synaptic density (Haug and Eggers, 1991), white matter integrity (Davis et al., 2009), descending inputs (Yue et al., 1999), changes in the musculoskeletal system (McGibbon, 2003), and degenerative changes in the central and peripheral nervous systems (Skinner et al., 1984; Seidler et al., 2010). The resulting locomotor changes characteristic of ageing are captured by the spinal topography (Figure 4B). As compared to young adults, the sacral motoneurons activation is significantly wider in older adults and shifts toward an earlier midstance, consistent with wider activity profiles of the muscles innervated by the sacral segments (Monaco et al., 2010). In addition, the occurrence of the maximal activation of the lumbar segments occurs significantly earlier in older than in young adults. This is related to the loss of anticipated COM step-to-step transition strategy in elderly (Figure 4B). Because of the lack of late push-off prior to toe-off from the trailing leg, the down-to-up redirection of the COM velocity starts later in older than in young adults (Dewolf et al., 2021).

In conclusion, with respect to the overall influence of age on the neuromechanics of motor pool activity (Figure 4), only in young adults do we observe the two features of motoneuron activation that are associated with the most energy-efficient bipedal gait: 1) distinct bursts of activity in the lumbar and sacral segments at the beginning and end of stance, respectively, and 2) their short durations relative to gait cycle duration. The next section discusses how patients with locomotor impairments may benefit from the restoration of a normal motor pool activity pattern, which may be related to the optimal pattern of spinal maps just summarized.

## Spinal maps for evaluating and neuromodulating disease-related spinal locomotor output

Differences in the spinal locomotor output are expected for patients who display impaired muscle activity. In recent years, significant advancements have been made in the development of multifaceted neurotechnologies, such as wearable powered lower limb exoskeletons, functional electrical stimulation of muscles, and spinal cord neuromodulation therapies to aid in walking and promote motor recovery. The growing interest to restoring and enhancing gait control in individuals with neurological disabilities (e.g., spinal cord injury) by applying new technologies has led a number of researchers to use spinal topography for investigating the spinal locomotor circuit impairments (Grasso et al., 2004; Scivoletto et al., 2007; Wagner et al., 2018; Hofstoetter et al., 2021; Zhvansky et al., 2022). Spinal maps can be used for both monitoring aberrant motor pool activation and neuromodulating the spinal locomotor circuitry.

**FIGURE 5:**
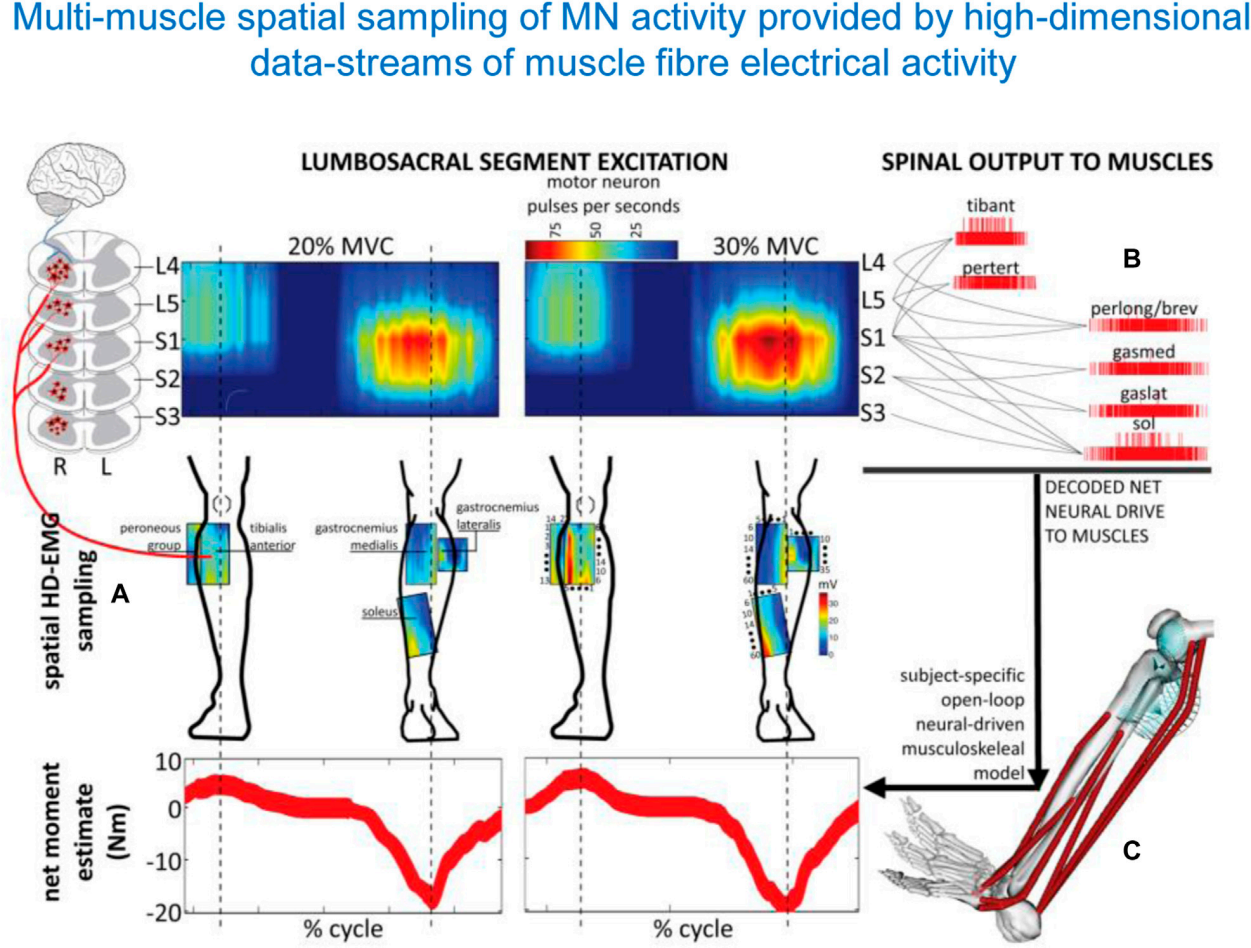
Spatiotemporal patterns of ipsilateral α-motoneuron activity in the spinal cord assessed by multi-muscle spatial sampling of high-density electromyograms (HD-EMG). The HD-EMG is decomposed to determine cumulative spike trains of the MNs **(A)** mapped onto the rostrocaudal axis of the spinal cord lumbosacral segments **(B)**. MN spike trains drive forward an open-loop subject-specific musculoskeletal model **(C)**. This method shows how the neural drive to muscles is generated by the spinal cord output layers, enabling ankle joint mechanical moment control across different forces (from Sartori et al., 2017).

The usage of the spinal maps of MN activation can be coupled with complementary statistical analysis of muscle synergies. They often converge to a similar temporal structure of the locomotor program consisting in a burst-like activation of groups of muscles, though the spinal maps additionally reflect the spatial loci of this activity in the spinal cord (Ivanenko et al., 2008; Yokoyama et al., 2017). Spinal maps and accurate α-motoneuron discharges across lumbosacral segments in the human spinal cord can also be assessed by employing multi-muscle spatial sampling and deconvolution of high-density muscle fibre electrical activity via 256 EMG channels simultaneously (Figure 5) (Sartori et al., 2017; Gogeascoechea et al., 2020). This enables observing causal associations between spinal motor neuron activity and joint moment control. While this review focusses on the lumbosacral enlargement innervating lower limb muscles during locomotion, the spinal maps can also be used to assess the motor pool activity patterns in the cervical spinal cord during both locomotor tasks and voluntary contractions, again by mapping the EMG activity profiles of the upper limb and trunk muscles onto the location of the motoneuron pools in the cervical enlargement (Grasso et al., 2004; Ivanenko et al., 2006; Pellegrino et al., 2021). Given the coupling between the cervical cord controlling arm muscles and the lumbosacral cord controlling leg muscles (involvement of the upper limbs, specifically the proximal arm muscles during walking, La Scaleia et al. (2014b), improvements in arm-leg coordination and adequate spatiotemporal neuromodulation of cervical motor pool activity may help ameliorating abnormal gait and upper limb and trunk control (Keller et al., 2021; Kaneshige et al., 2022; Oh et al., 2022). For instance, cervical stimulation of specific segments can potentiate locomotor output in the lower limbs, likely via augmentation of descending drive and/or descending propriospinal system (Gerasimenko Y. et al., 2015; Barss et al., 2020).

**FIGURE 6:**
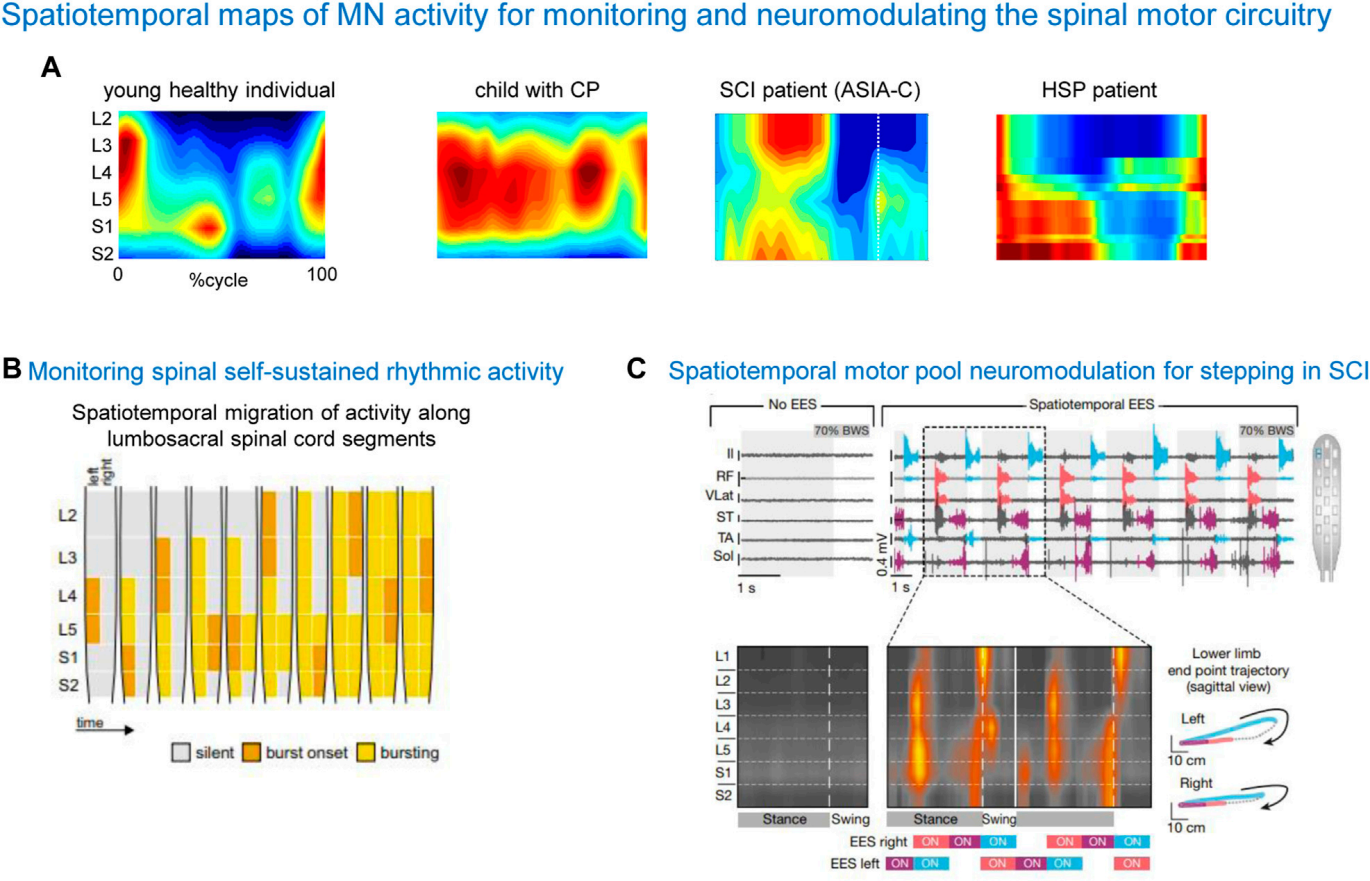
Spatiotemporal maps of α-MN activity for monitoring and neuromodulating the spinal motor circuitry. **(A)** Examples of spinal activity maps in a typically developing child, child with cerebral palsy (CP, from Cappellini et al., 2016), spinal cord injury patient (SCI, from Ivanenko et al., 2009) and patient with hereditary spastic paraplegia (HSP, from Martino et al., 2018). Note an impaired lumbar-to-sacral oscillation of motor pool activity during the stance phase in pathological cases. **(B)** An example of using spinal maps for monitoring spinal self-sustained rhythmic activity in SCI (adapted from Minassian et al., 2023, with permission from Springer Nature). **(C)** An example of using spinal maps for designing the spatiotemporal configuration of epidural electrical stimulation (EES) and monitoring segmental activity in SCI patients (adapted from Wagner et al., 2018, with permission from Springer Nature).

One rationale of using spinal maps in patients with locomotor deficits is to evaluate the naturalness or similarities of the neural control strategy in patients with respect to normal walking, since an abnormal spatiotemporal integration of spinal motor activity may result in a risk for failure or abnormalities in gait recovery. Also, an important aspect for developing spinal cord modulating therapies is the physiologically relevant assessment of the state of the spinal circuitry. Figure 6A shows some examples of notable impairments of spinal motoneuron activity maps in locomotor-related disorders such as cerebral palsy (CP), spinal cord injury (SCI), and hereditary spastic paraplegia (HSP). Notice an impaired lumbar-to-sacral oscillation pattern during the stance phase in all illustrated pathological gaits. As described above, the main feature of the spinal maps in neurologically intact individuals is a prominent separation of the lumbar and sacral activation bursts (Figure 6A, left panel). In contrast, the prominent feature of the maps in a child with CP is a quasi-synchronous involvement of lumbar and sacral segments during stance and a wider motor pool activation during stance, reminiscent of the maps of toddlers at the onset of independent walking (Cappellini et al., 2016). In SCI patients, the lumbosacral enlargement often shows abnormal loci of activation, such as an upper lumbar segment activation at midstance (Ivanenko et al., 2009) (Figure 5A). In HSP patients, the activity timings in lumbar and sacral segments tend to be quasi-synchronous, because of a typical progressive widening of the activity involving the sacral segments (Martino et al., 2018). A widening of spinal locomotor output spreading from caudal to rostral segments is associated with the degeneration of the corticospinal tract in HSP. These findings highlight pathophysiologically relevant differential changes in the spinal locomotor output in HSP related to the specific innervation of muscles in the spinal cord. Abnormal spatiotemporal segregation of spinal motor activity during gait is likewise observed in other populations, for instance, in stroke patients (Coscia et al., 2015). Spinal maps have been also used to assess self-sustained rhythmic spinal myoclonus in the paralyzed lower limbs based on the segmental innervations of the recruited muscles (Minassian et al., 2023), showing a complex migration of activity along the lumbar and upper sacral spinal cord (Figure 6B). Thus, spinal maps might be helpful for identifying patient-specific physiological markers of the disease, and developing targeted therapeutic strategies.

Another rationale for considering the spinal maps of motor pool activity is offered by the promising strategy of neuromodulating the spinal circuitry using spinal cord electrical stimulation (SCES). We have previously argued that the temporal patterns of MN activation correlate with global kinematic goals of locomotion, and that they could be potentially useful to drive neuroprostheses for SCI patients that utilize spatially distributed stimulators (Ivanenko et al., 2003). Recent research has effectively used this approach using either epidural (Angeli et al., 2018; Hofstoetter et al., 2021; Rowald et al., 2022; Mesbah et al., 2023) or transcutaneous (Gerasimenko Y. P. et al., 2015; Sayenko et al., 2015; Solopova et al., 2017; Siu et al., 2022; Bryson et al., 2023) multi-segmental SCES to restore leg motor functions. SCES indirectly activates motor neurons pools located in the spinal cord segment by recruiting proprioceptive circuits within the posterior roots of the spinal cord. Figure 6C shows a successful application of SCES based on the spinal maps in a complete SCI patient. The mechanism underlying neurostimulation strategies (Wagner et al., 2018) consists of stimulating the expected hotspots of MN activation, ensuring weight acceptance, propulsion and swing. After identifying electrodes position that should target specific subsets of dorsal roots projecting to the spinal cord regions associated with extensor or flexor hot spots, targeted SCES effectively activated the regions embedding these hotspots, adjusting the onset and duration of each stimulation train to approach the motor neuron activation maps of healthy individuals (Wagner et al., 2018). These SCES stimulation sequences enabled robust EMG activity in otherwise quiescent muscles of a patient with SCI during stepping on a treadmill (Figure 6C). Combining SCES with exoskeleton gait training can have a synergistic rehabilitative effect on restoring walking abilities, somatic sensation, and cardiovascular and bladder function in paralyzed individuals (Ivanenko et al., 2023). SCES enables the modulation of specific leg motor pools (Capogrosso et al., 2013; Wenger et al., 2014; Moraud et al., 2016; Wenger et al., 2016), and restores standing and basic walking in human with paralysis due to SCI (Minassian et al., 2004; Angeli et al., 2014; Angeli et al., 2018; Formento et al., 2018; Gill et al., 2018) and also in animals (Wenger et al., 2014; Capogrosso et al., 2016; Wenger et al., 2016; Asboth et al., 2018; Bonizzato et al., 2021), improving the capacity of the spinal cord to convert task-specific sensory information into the locomotor-related muscle activity. Additionally, spatiotemporal SCES is now included into brain-spine interfaces for walking (Lorach et al., 2023).

These findings underlie the practical importance of the spatiotemporal activity patterns of motoneurons as causal drives leading to clinical improvements. Thus, it has been shown that an optimal design of the location and timing of epidural SCES tailored on the individual features of spinal cord topology so as to reproduce the natural spatiotemporal activation of motoneurons leads to successful rehabilitation of spinal-cord injured patients with complete paralysis (Rowald et al., 2022).

The comprehensive understanding of the functional spinal maps and neural circuits can pave the way for developing targeted therapeutic strategies and contribute to the advancement of neuroprosthetics aimed at restoring functional movement patterns in patients with locomotor deficits. To develop clinical treatments, a range of factors should be considered, including abnormalities in the spinal activity maps, individual differences in spinal cord anatomy, physiology, dosing, personalized CPG-modulating therapies, as well as long-term effects of this treatment. All these factors share the same goal: activating and remodelling the spinal neuronal pathways to get closer to the typical evolutionary evolved neuromechanics of spinal locomotor circuitry functioning. The spinal maps may also have an important application in the emerging field of interpersonal coordination for interactive locomotion (Sylos-Labini et al., 2018).

## References

[B1] Alexander R. M. (1991). Energy-saving mechanisms in walking and running. J. Exp. Biol. 160, 55–69. 10.1242/jeb.160.1.55 1960518

[B2] Angeli C. A. Boakye M. Morton R. A. Vogt J. Benton K. Chen Y. (2018). Recovery of over-ground walking after chronic motor complete spinal cord injury. N. Engl. J. Med. 379, 1244–1250. 10.1056/NEJMoa1803588 30247091

[B3] Angeli C. A. Edgerton V. R. Gerasimenko Y. P. Harkema S. J. (2014). Altering spinal cord excitability enables voluntary movements after chronic complete paralysis in humans. Brain 137, 1394–1409. 10.1093/brain/awu038 24713270 PMC3999714

[B4] Arber S. (2012). Motor circuits in action: specification, connectivity, and function. Neuron 74, 975–989. 10.1016/j.neuron.2012.05.011 22726829

[B5] Asboth L. Friedli L. Beauparlant J. Martinez-Gonzalez C. Anil S. Rey E. (2018). Cortico-reticulo-spinal circuit reorganization enables functional recovery after severe spinal cord contusion. Nat. Neurosci. 21, 576–588. 10.1038/s41593-018-0093-5 29556028

[B6] Ausborn J. Snyder A. C. Shevtsova N. A. Rybak I. A. Rubin J. E. (2018). State-dependent rhythmogenesis and frequency control in a half-center locomotor CPG. J. Neurophysiol. 119, 96–117. 10.1152/jn.00550.2017 28978767 PMC5866471

[B7] Barkan C. L. Zornik E. (2019). Feedback to the future: motor neuron contributions to central pattern generator function. J. Exp. Biol. 222, jeb193318. 10.1242/jeb.193318 31420449 PMC6739810

[B8] Barss T. S. Parhizi B. Mushahwar V. K. (2020). Transcutaneous spinal cord stimulation of the cervical cord modulates lumbar networks. J. Neurophysiol. 123, 158–166. 10.1152/jn.00433.2019 31747338

[B9] Bhumbra G. S. Beato M. (2018). Recurrent excitation between motoneurones propagates across segments and is purely glutamatergic. PLoS Biol. 16, e2003586. 10.1371/journal.pbio.2003586 29538375 PMC5851534

[B10] Bonizzato M. James N. D. Pidpruzhnykova G. Pavlova N. Shkorbatova P. Baud L. (2021). Multi-pronged neuromodulation intervention engages the residual motor circuitry to facilitate walking in a rat model of spinal cord injury. Nat. Commun. 12, 1925. 10.1038/s41467-021-22137-9 33771986 PMC7997909

[B11] Bramble D. M. Lieberman D. E. (2004). Endurance running and the evolution of Homo. Nature 432, 345–352. 10.1038/nature03052 15549097

[B12] BrysonN.LombardiL.HawthornR.FeiJ.KeeseyR.PeifferJ. D. (2023). Enhanced selectivity of transcutaneous spinal cord stimulation by multielectrode configuration. J. Neural Eng. 20, 046015. 10.1088/1741-2552/ace552 PMC1048138737419109

[B13] Buchanan J. T. Grillner S. (1987). Newly identified “glutamate interneurons” and their role in locomotion in the lamprey spinal cord. Science 236, 312–314. 10.1126/science.3563512 3563512

[B14] Capogrosso M. Milekovic T. Borton D. Wagner F. Moraud E. M. Mignardot J.-B. (2016). A brain-spine interface alleviating gait deficits after spinal cord injury in primates. Nature 539, 284–288. 10.1038/nature20118 27830790 PMC5108412

[B15] Capogrosso M. Wenger N. Raspopovic S. Musienko P. Beauparlant J. Bassi Luciani L. (2013). A computational model for epidural electrical stimulation of spinal sensorimotor circuits. J. Neurosci. 33, 19326–19340. 10.1523/JNEUROSCI.1688-13.2013 24305828 PMC6618777

[B16] Cappellini G. Ivanenko Y. P. Dominici N. Poppele R. E. Lacquaniti F. (2010). Migration of motor pool activity in the spinal cord reflects body mechanics in human locomotion. J. Neurophysiol. 104, 3064–3073. 10.1152/jn.00318.2010 20881204

[B17] Cappellini G. Ivanenko Y. P. Martino G. MacLellan M. J. Sacco A. Morelli D. (2016). Immature spinal locomotor output in children with cerebral palsy. Front. Physiol. 7, 478. 10.3389/fphys.2016.00478 27826251 PMC5078720

[B18] Cappellini G. Ivanenko Y. P. Poppele R. E. Lacquaniti F. (2006). Motor patterns in human walking and running. J. Neurophysiol. 95, 3426–3437. 10.1152/jn.00081.2006 16554517

[B19] Cazalets J. R. Bertrand S. (2000). Ubiquity of motor networks in the spinal cord of vertebrates. Brain Res. Bull. 53, 627–634. 10.1016/s0361-9230(00)00396-8 11165798

[B20] Chopek J. W. Nascimento F. Beato M. Brownstone R. M. Zhang Y. (2018). Sub-populations of spinal V3 interneurons form focal modules of layered pre-motor microcircuits. Cell Rep. 25, 146–156. 10.1016/j.celrep.2018.08.095 30282024 PMC6180347

[B21] Coscia M. Monaco V. Martelloni C. Rossi B. Chisari C. Micera S. (2015). Muscle synergies and spinal maps are sensitive to the asymmetry induced by a unilateral stroke. J. Neuroeng Rehabil. 12, 39. 10.1186/s12984-015-0031-7 25928264 PMC4411739

[B22] Davis S. W. Dennis N. A. Buchler N. G. White L. E. Madden D. J. Cabeza R. (2009). Assessing the effects of age on long white matter tracts using diffusion tensor tractography. Neuroimage 46, 530–541. 10.1016/j.neuroimage.2009.01.068 19385018 PMC2775533

[B23] de Sèze M. Falgairolle M. Viel S. Assaiante C. Cazalets J.-R. (2008). Sequential activation of axial muscles during different forms of rhythmic behavior in man. Exp. Brain Res. 185, 237–247. 10.1007/s00221-007-1146-2 17940760

[B24] Dewolf A. H. Ivanenko Y. P. Zelik K. E. Lacquaniti F. Willems P. A. (2019). Differential activation of lumbar and sacral motor pools during walking at different speeds and slopes. J. Neurophysiol. 122, 872–887. 10.1152/jn.00167.2019 31291150 PMC6734402

[B25] DewolfA. H.Sylos-LabiniF.CappelliniG.ZhvanskyD.WillemsP. A.IvanenkoY. (2021). Neuromuscular age-related adjustment of gait when moving upwards and downwards. Front. Hum. Neurosci. 15, 749366. 10.3389/fnhum.2021.749366 34744664 PMC8566537

[B26] Doherty T. J. (2003). Invited review: aging and sarcopenia. J. Appl. Physiol. 95, 1717–1727. 10.1152/japplphysiol.00347.2003 12970377

[B27] Falgairolle M. de Seze M. Juvin L. Morin D. Cazalets J.-R. (2006). Coordinated network functioning in the spinal cord: an evolutionary perspective. J. Physiology-Paris 100, 304–316. 10.1016/j.jphysparis.2007.05.003 17658245

[B28] Formento E. Minassian K. Wagner F. Mignardot J. B. Le Goff-Mignardot C. G. Rowald A. (2018). Electrical spinal cord stimulation must preserve proprioception to enable locomotion in humans with spinal cord injury. Nat. Neurosci. 21, 1728–1741. 10.1038/s41593-018-0262-6 30382196 PMC6268129

[B29] Gerasimenko Y. Gorodnichev R. Machueva E. Pivovarova E. Semyenov D. Savochin A. (2010). Novel and direct access to the human locomotor spinal circuitry. J. Neurosci. 30, 3700–3708. 10.1523/JNEUROSCI.4751-09.2010 20220003 PMC2847395

[B30] Gerasimenko Y. Gorodnichev R. Puhov A. Moshonkina T. Savochin A. Selionov V. (2015a). Initiation and modulation of locomotor circuitry output with multisite transcutaneous electrical stimulation of the spinal cord in noninjured humans. J. Neurophysiol. 113, 834–842. 10.1152/jn.00609.2014 25376784

[B31] Gerasimenko Y. P. Lu D. C. Modaber M. Zdunowski S. Gad P. Sayenko D. G. (2015b). Noninvasive reactivation of motor descending control after paralysis. J. Neurotrauma 32, 1968–1980. 10.1089/neu.2015.4008 26077679 PMC4677519

[B32] Gill M. L. Grahn P. J. Calvert J. S. Linde M. B. Lavrov I. A. Strommen J. A. (2018). Neuromodulation of lumbosacral spinal networks enables independent stepping after complete paraplegia. Nat. Med. 24, 1677–1682. 10.1038/s41591-018-0175-7 30250140

[B33] Giszter S. Patil V. Hart C. (2007). Primitives, premotor drives, and pattern generation: a combined computational and neuroethological perspective. Prog. Brain Res. 165, 323–346. 10.1016/S0079-6123(06)65020-6 17925255

[B34] Gogeascoechea A. Kuck A. van Asseldonk E. Negro F. Buitenweg J. R. Yavuz U. S. (2020). Interfacing with alpha motor neurons in spinal cord injury patients receiving trans-spinal electrical stimulation. Front. Neurol. 11, 493. 10.3389/fneur.2020.00493 32582012 PMC7296155

[B35] Good C. D. Johnsrude I. S. Ashburner J. Henson R. N. Friston K. J. Frackowiak R. S. (2001). A voxel-based morphometric study of ageing in 465 normal adult human brains. Neuroimage 14, 21–36. 10.1006/nimg.2001.0786 11525331

[B36] Grasso R. Ivanenko Y. P. Zago M. Molinari M. Scivoletto G. Castellano V. (2004). Distributed plasticity of locomotor pattern generators in spinal cord injured patients. Brain 127, 1019–1034. 10.1093/brain/awh115 14988161

[B37] Grillner S. (1981). “Control of locomotion in bipeds, tetrapods and fish,” in Handbook of physiology: section 1: the nervous system, volume II, Part.1 motor control (Bethesda, MD: Wiley), 1179–1236.

[B38] Grillner S. (2018). Evolution: vertebrate limb control over 420 million years. Curr. Biol. 28, R162–R164. 10.1016/j.cub.2017.12.040 29462584

[B39] Grillner S. El Manira A. (2015). The intrinsic operation of the networks that make us locomote. Curr. Opin. Neurobiol. 31, 244–249. 10.1016/j.conb.2015.01.003 25599926

[B40] Grillner S. El Manira A. (2020). Current principles of motor control, with special reference to vertebrate locomotion. Physiol. Rev. 100, 271–320. 10.1152/physrev.00015.2019 31512990

[B41] GrillnerS.KozlovA. (2021). The CPGs for limbed locomotion-facts and fiction. Int. J. Mol. Sci. 22, 5882. 10.3390/ijms22115882 34070932 PMC8198624

[B42] Hanson M. G. Landmesser L. T. (2003). Characterization of the circuits that generate spontaneous episodes of activity in the early embryonic mouse spinal cord. J. Neurosci. 23, 587–600. 10.1523/JNEUROSCI.23-02-00587.2003 12533619 PMC6741864

[B43] Haug H. Eggers R. (1991). Morphometry of the human cortex cerebri and corpus striatum during aging. Neurobiol. Aging 12, 336–338. 10.1016/0197-4580(91)90013-a 1961364

[B44] Hemmerling K. J. Hoggarth M. A. Sandhu M. S. Parrish T. B. Bright M. G. (2023). Spatial distribution of hand-grasp motor task activity in spinal cord functional magnetic resonance imaging. Hum. Brain Mapp. 44, 5567–5581. 10.1002/hbm.26458 37608682 PMC10619382

[B45] Henderson G. Tomlinson B. E. Gibson P. H. (1980). Cell counts in human cerebral cortex in normal adults throughout life using an image analysing computer. J. Neurol. Sci. 46, 113–136. 10.1016/0022-510x(80)90048-9 7373341

[B46] Hofstoetter U. S. Perret I. Bayart A. Lackner P. Binder H. Freundl B. (2021). Spinal motor mapping by epidural stimulation of lumbosacral posterior roots in humans. iScience 24, 101930. 10.1016/j.isci.2020.101930 33409476 PMC7773960

[B47] HsuL.-J.BerthoM.KiehnO. (2023). Deconstructing the modular organization and real-time dynamics of mammalian spinal locomotor networks. Nat. Commun. 14, 873. 10.1038/s41467-023-36587-w 36797254 PMC9935527

[B48] Ivanenko Y. Shapkova E. Y. Petrova D. A. Kleeva D. F. Lebedev M. A. (2023). Exoskeleton gait training with spinal cord neuromodulation. Front. Hum. Neurosci. 17, 1194702. 10.3389/fnhum.2023.1194702 37250689 PMC10213721

[B49] Ivanenko Y. P. Cappellini G. Poppele R. E. Lacquaniti F. (2008). Spatiotemporal organization of alpha-motoneuron activity in the human spinal cord during different gaits and gait transitions. Eur. J. Neurosci. 27, 3351–3368. 10.1111/j.1460-9568.2008.06289.x 18598271

[B50] IvanenkoY. P.DominiciN.CappelliniG.Di PaoloA.GianniniC.PoppeleR. E. (2013). Changes in the spinal segmental motor output for stepping during development from infant to adult. J. Neurosci. 33, 3025–336a. 10.1523/JNEUROSCI.2722-12.2013 23407959 PMC6619203

[B51] Ivanenko Y. P. Grasso R. Zago M. Molinari M. Scivoletto G. Castellano V. (2003). Temporal components of the motor patterns expressed by the human spinal cord reflect foot kinematics. J. Neurophysiol. 90, 3555–3565. 10.1152/jn.00223.2003 12853436

[B52] Ivanenko Y. P. Poppele R. E. Lacquaniti F. (2004). Five basic muscle activation patterns account for muscle activity during human locomotion. J. Physiol. (Lond.) 556, 267–282. 10.1113/jphysiol.2003.057174 14724214 PMC1664897

[B53] Ivanenko Y. P. Poppele R. E. Lacquaniti F. (2006). Spinal cord maps of spatiotemporal alpha-motoneuron activation in humans walking at different speeds. J. Neurophysiol. 95, 602–618. 10.1152/jn.00767.2005 16282202

[B54] Ivanenko Y. P. Poppele R. E. Lacquaniti F. (2009). Distributed neural networks for controlling human locomotion: lessons from normal and SCI subjects. Brain Res. Bull. 78, 13–21. 10.1016/j.brainresbull.2008.03.018 19070781

[B55] Jenny A. B. Inukai J. (1983). Principles of motor organization of the monkey cervical spinal cord. J. Neurosci. 3, 567–575. 10.1523/JNEUROSCI.03-03-00567.1983 6827309 PMC6564558

[B56] Jessell T. M. Sürmeli G. Kelly J. S. (2011). Motor neurons and the sense of place. Neuron 72, 419–424. 10.1016/j.neuron.2011.10.021 22078502

[B57] Kaneshige M. Obara K. Suzuki M. Tazoe T. Nishimura Y. (2022). Tuning of motor outputs produced by spinal stimulation during voluntary control of torque directions in monkeys. Elife 11, e78346. 10.7554/eLife.78346 36512395 PMC9747157

[B58] KaptanM.HornU.VannesjoS. J.MildnerT.WeiskopfN.FinsterbuschJ. (2023). Reliability of resting-state functional connectivity in the human spinal cord: assessing the impact of distinct noise sources. Neuroimage 275, 120152. 10.1016/j.neuroimage.2023.120152 37142169 PMC10262064

[B59] KellerA.SinghG.SommerfeldJ. H.KingM.ParikhP.UgiliwenezaB. (2021). Noninvasive spinal stimulation safely enables upright posture in children with spinal cord injury. Nat. Commun. 12, 5850. 10.1038/s41467-021-26026-z 34615867 PMC8494794

[B60] Kendall F. McCreary E. Provance P. Rodgers M. Romani W. (2005) Muscles. Testing and function with posture and pain. Baltimore: Lippincott Williams and Wilkins.

[B61] Kiehn O. (2006). Locomotor circuits in the mammalian spinal cord. Annu. Rev. Neurosci. 29, 279–306. 10.1146/annurev.neuro.29.051605.112910 16776587

[B62] Kiehn O. (2016). Decoding the organization of spinal circuits that control locomotion. Nat. Rev. Neurosci. 17, 224–238. 10.1038/nrn.2016.9 26935168 PMC4844028

[B63] Kinany N. Khatibi A. Lungu O. Finsterbusch J. Büchel C. Marchand-Pauvert V. (2023). Decoding cerebro-spinal signatures of human behavior: application to motor sequence learning. Neuroimage 275, 120174. 10.1016/j.neuroimage.2023.120174 37201642

[B64] Kornelsen J. Stroman P. W. (2004). fMRI of the lumbar spinal cord during a lower limb motor task. Magn. Reson Med. 52, 411–414. 10.1002/mrm.20157 15282826

[B65] Kornelsen J. Stroman P. W. (2007). Detection of the neuronal activity occurring caudal to the site of spinal cord injury that is elicited during lower limb movement tasks. Spinal Cord. 45, 485–490. 10.1038/sj.sc.3102019 17245349

[B66] Lacquaniti F. Grasso R. Zago M. (1999). Motor patterns in walking. News Physiol. Sci. 14, 168–174. 10.1152/physiologyonline.1999.14.4.168 11390844

[B67] Lacquaniti F. Ivanenko Y. P. Zago M. (2012). Patterned control of human locomotion. J. Physiol. (Lond.) 590, 2189–2199. 10.1113/jphysiol.2011.215137 22411012 PMC3424743

[B68] Landelle C. Lungu O. Vahdat S. Kavounoudias A. Marchand-Pauvert V. De Leener B. (2021). Investigating the human spinal sensorimotor pathways through functional magnetic resonance imaging. Neuroimage 245, 118684. 10.1016/j.neuroimage.2021.118684 34732324

[B69] La Scaleia V. Ivanenko Y. P. Zelik K. E. Lacquaniti F. (2014a). Spinal motor outputs during step-to-step transitions of diverse human gaits. Front. Hum. Neurosci. 8, 305. 10.3389/fnhum.2014.00305 24860484 PMC4030139

[B70] La Scaleia V. Sylos-Labini F. Hoellinger T. Wang L. Cheron G. Lacquaniti F. (2014b). Control of leg movements driven by EMG activity of shoulder muscles. Front. Hum. Neurosci. 8, 838. 10.3389/fnhum.2014.00838 25368569 PMC4202724

[B71] Lawrence J. M. Stroman P. W. Kollias S. S. (2008). Functional magnetic resonance imaging of the human spinal cord during vibration stimulation of different dermatomes. Neuroradiology 50, 273–280. 10.1007/s00234-007-0338-6 18026942

[B72] Lev-Tov A. Delvolvé I. Kremer E. (2000). Sacrocaudal afferents induce rhythmic efferent bursting in isolated spinal cords of neonatal rats. J. Neurophysiol. 83, 888–894. 10.1152/jn.2000.83.2.888 10669502

[B73] Lorach H. Galvez A. Spagnolo V. Martel F. Karakas S. Intering N. (2023). Walking naturally after spinal cord injury using a brain-spine interface. Nature 618, 126–133. 10.1038/s41586-023-06094-5 37225984 PMC10232367

[B74] MachadoT. A.PnevmatikakisE.PaninskiL.JessellT. M.MiriA. (2015). Primacy of flexor locomotor pattern revealed by ancestral reversion of motor neuron identity. Cell 162, 338–350. 10.1016/j.cell.2015.06.036 26186188 PMC4540486

[B75] Maki B. E. (1997). Gait changes in older adults: predictors of falls or indicators of fear. J. Am. Geriatr. Soc. 45, 313–320. 10.1111/j.1532-5415.1997.tb00946.x 9063277

[B76] Marder E. (1991). Modifiability of pattern generation. Curr. Opin. Neurobiol. 1, 571–576. 10.1016/s0959-4388(05)80030-3 1822298

[B77] Martino G. Ivanenko Y. Serrao M. Ranavolo A. Draicchio F. Rinaldi M. (2018). Differential changes in the spinal segmental locomotor output in Hereditary Spastic Paraplegia. Clin. Neurophysiol. 129, 516–525. 10.1016/j.clinph.2017.11.028 29353180

[B78] McGibbon C. A. (2003). Toward a better understanding of gait changes with age and disablement: neuromuscular adaptation. Exerc Sport Sci. Rev. 31, 102–108. 10.1097/00003677-200304000-00009 12715975

[B79] Mentis G. Z. Alvarez F. J. Bonnot A. Richards D. S. Gonzalez-Forero D. Zerda R. (2005). Noncholinergic excitatory actions of motoneurons in the neonatal mammalian spinal cord. Proc. Natl. Acad. Sci. U. S. A. 102, 7344–7349. 10.1073/pnas.0502788102 15883359 PMC1091756

[B80] Mesbah S. Herrity A. Ugiliweneza B. Angeli C. Gerasimenko Y. Boakye M. (2023). Neuroanatomical mapping of the lumbosacral spinal cord in individuals with chronic spinal cord injury. Brain Commun. 5, fcac330. 10.1093/braincomms/fcac330 36632181 PMC9825531

[B81] Minassian K. Bayart A. Lackner P. Binder H. Freundl B. Hofstoetter U. S. (2023). Rare phenomena of central rhythm and pattern generation in a case of complete spinal cord injury. Nat. Commun. 14, 3276. 10.1038/s41467-023-39034-y 37280242 PMC10244420

[B82] Minassian K. Jilge B. Rattay F. Pinter M. M. Binder H. Gerstenbrand F. (2004). Stepping-like movements in humans with complete spinal cord injury induced by epidural stimulation of the lumbar cord: electromyographic study of compound muscle action potentials. Spinal Cord. 42, 401–416. 10.1038/sj.sc.3101615 15124000

[B83] Monaco V. Ghionzoli A. Micera S. (2010). Age-related modifications of muscle synergies and spinal cord activity during locomotion. J. Neurophysiol. 104, 2092–2102. 10.1152/jn.00525.2009 20685924

[B84] Moraud E. M. Capogrosso M. Formento E. Wenger N. DiGiovanna J. Courtine G. (2016). Mechanisms underlying the neuromodulation of spinal circuits for correcting gait and balance deficits after spinal cord injury. Neuron 89, 814–828. 10.1016/j.neuron.2016.01.009 26853304

[B85] Neptune R. R. Clark D. J. Kautz S. A. (2009). Modular control of human walking: a simulation study. J. Biomech. 42, 1282–1287. 10.1016/j.jbiomech.2009.03.009 19394023 PMC2696580

[B86] O’Donovan M. J. Wenner P. Chub N. Tabak J. Rinzel J. (1998). Mechanisms of spontaneous activity in the developing spinal cord and their relevance to locomotion. Ann. N. Y. Acad. Sci. 860, 130–141. 10.1111/j.1749-6632.1998.tb09044.x 9928307

[B87] Oh J. Steele A. G. Varghese B. Martin C. A. Scheffler M. S. Markley R. L. (2022). Cervical transcutaneous spinal stimulation for spinal motor mapping. iScience 25, 105037. 10.1016/j.isci.2022.105037 36147963 PMC9485062

[B88] Pellegrino L. Coscia M. Giannoni P. Marinelli L. Casadio M. (2021). Stroke impairs the control of isometric forces and muscle activations in the ipsilesional arm. Sci. Rep. 11, 18533. 10.1038/s41598-021-96329-0 34535693 PMC8448776

[B89] Pham B. N. Luo J. Anand H. Kola O. Salcedo P. Nguyen C. (2020). Redundancy and multifunctionality among spinal locomotor networks. J. Neurophysiol. 124, 1469–1479. 10.1152/jn.00338.2020 32966757 PMC8356786

[B90] Powers J. M. Ioachim G. Stroman P. W. (2018). Ten key insights into the use of spinal cord fMRI. Brain Sci. 8, 173. 10.3390/brainsci8090173 30201938 PMC6162663

[B91] Romanes G. J. (1941). The development and significance of the cell columns in the ventral horn of the cervical and upper thoracic spinal cord of the rabbit. J. Anat. 76, 112–130. 17104877 PMC1252719

[B92] Romanes G. J. (1951). The motor cell columns of the lumbo-sacral spinal cord of the cat. J. Comp. Neurol. 94, 313–363. 10.1002/cne.900940209 14832391

[B93] Romanes G. J. (1964). The motor pools of the spinal cord. Prog. Brain Res. 11, 93–119. 10.1016/s0079-6123(08)64045-5 14300484

[B94] Routal R. V. Pal G. P. (1999). A study of motoneuron groups and motor columns of the human spinal cord. J. Anat. 195 (2), 211–224. 10.1046/j.1469-7580.1999.19520211.x 10529058 PMC1467986

[B95] Rowald A. Komi S. Demesmaeker R. Baaklini E. Hernandez-Charpak S. D. Paoles E. (2022). Activity-dependent spinal cord neuromodulation rapidly restores trunk and leg motor functions after complete paralysis. Nat. Med. 28, 260–271. 10.1038/s41591-021-01663-5 35132264

[B96] Sartori M. Yavuz U. Ş. Farina D. (2017). *In vivo* neuromechanics: decoding causal motor neuron behavior with resulting musculoskeletal function. Sci. Rep. 7, 13465. 10.1038/s41598-017-13766-6 29044165 PMC5647446

[B97] Sayenko D. G. Atkinson D. A. Dy C. J. Gurley K. M. Smith V. L. Angeli C. (2015). Spinal segment-specific transcutaneous stimulation differentially shapes activation pattern among motor pools in humans. J. Appl. Physiol. 118, 1364–1374. 10.1152/japplphysiol.01128.2014 25814642 PMC4451290

[B98] Scivoletto G. Ivanenko Y. Morganti B. Grasso R. Zago M. Lacquaniti F. (2007). Plasticity of spinal centers in spinal cord injury patients: new concepts for gait evaluation and training. Neurorehabil Neural Repair 21, 358–365. 10.1177/1545968306295561 17353461

[B99] Seidler R. D. Bernard J. A. Burutolu T. B. Fling B. W. Gordon M. T. Gwin J. T. (2010). Motor control and aging: links to age-related brain structural, functional, and biochemical effects. Neurosci. Biobehav Rev. 34, 721–733. 10.1016/j.neubiorev.2009.10.005 19850077 PMC2838968

[B100] Selionov V. A. Ivanenko Y. P. Solopova I. A. Gurfinkel V. S. (2009). Tonic central and sensory stimuli facilitate involuntary air-stepping in humans. J. Neurophysiol. 101, 2847–2858. 10.1152/jn.90895.2008 19339461

[B101] Sharrard W. J. (1955). The distribution of the permanent paralysis in the lower limb in poliomyelitis; a clinical and pathological study. J. Bone Jt. Surg. Br. 37-B, 540–558. 10.1302/0301-620X.37B4.540 13271481

[B102] SharrardW. J. (1964). The segmental innervation of the lower limb muscles in man. Ann. R. Coll. Surg. Engl. 35, 106–122.14180405 PMC2311748

[B103] Sherrington C. (1906) The integrative action of the nervous system. New York: Charles Scribner’s Sons.

[B104] Siu R. Brown E. H. Mesbah S. Gonnelli F. Pisolkar T. Edgerton V. R. (2022). Novel noninvasive spinal neuromodulation strategy facilitates recovery of stepping after motor complete paraplegia. J. Clin. Med. 11, 3670. 10.3390/jcm11133670 35806954 PMC9267673

[B105] Skinner H. B. Barrack R. L. Cook S. D. (1984). Age-related decline in proprioception. Clin. Orthop. Relat. Res. 184, 208–211. 10.1097/00003086-198404000-00035 6705349

[B106] Smith J. L. Carlson-Kuhta P. Trank T. V. (1998). Forms of forward quadrupedal locomotion. III. A comparison of posture, hindlimb kinematics, and motor patterns for downslope and level walking. J. Neurophysiol. 79, 1702–1716. 10.1152/jn.1998.79.4.1702 9535940

[B107] Solopova I. A. Sukhotina I. A. Zhvansky D. S. Ikoeva G. A. Vissarionov S. V. Baindurashvili A. G. (2017). Effects of spinal cord stimulation on motor functions in children with cerebral palsy. Neurosci. Lett. 639, 192–198. 10.1016/j.neulet.2017.01.003 28063935

[B108] Song J. Ampatzis K. Björnfors E. R. El Manira A. (2016). Motor neurons control locomotor circuit function retrogradely via gap junctions. Nature 529, 399–402. 10.1038/nature16497 26760208

[B109] Stroman P. W. Wheeler-Kingshott C. Bacon M. Schwab J. M. Bosma R. Brooks J. (2014). The current state-of-the-art of spinal cord imaging: methods. Neuroimage 84, 1070–1081. 10.1016/j.neuroimage.2013.04.124 23685159 PMC4371133

[B110] Studenski S. Perera S. Patel K. Rosano C. Faulkner K. Inzitari M. (2011). Gait speed and survival in older adults. JAMA 305, 50–58. 10.1001/jama.2010.1923 21205966 PMC3080184

[B128] Sylos-LabiniF.d’AvellaA.LacquanitiF.IvanenkoY. (2018). Human-human interaction forces and interlimb coordination during side-by-side walking with hand contact. Front. Physiol. 9, 179. 10.3389/fphys.2018.00179 29563883 PMC5850283

[B111] Taga G. (1995). A model of the neuro-musculo-skeletal system for human locomotion. I. Emergence of basic gait. Biol. Cybern. 73, 97–111. 10.1007/BF00204048 7662771

[B112] Taitano R. I. Yakovenko S. Gritsenko V. (2024). Muscle anatomy is reflected in the spatial organization of the spinal motoneuron pools. Commun. Biol. 7, 97. 10.1038/s42003-023-05742-w 38225362 PMC10789783

[B113] Tomlinson B. E. Irving D. (1977). The numbers of limb motor neurons in the human lumbosacral cord throughout life. J. Neurol. Sci. 34, 213–219. 10.1016/0022-510x(77)90069-7 925710

[B114] Toossi A. Everaert D. G. Perlmutter S. I. Mushahwar V. K. (2019). Functional organization of motor networks in the lumbosacral spinal cord of non-human primates. Sci. Rep. 9, 13539. 10.1038/s41598-019-49328-1 31537819 PMC6753145

[B115] Vahdat S. Lungu O. Cohen-Adad J. Marchand-Pauvert V. Benali H. Doyon J. (2015). Simultaneous brain-cervical cord fMRI reveals intrinsic spinal cord plasticity during motor sequence learning. PLoS Biol. 13, e1002186. 10.1371/journal.pbio.1002186 26125597 PMC4488354

[B116] Vanderhorst V. G. Holstege G. (1997). Organization of lumbosacral motoneuronal cell groups innervating hindlimb, pelvic floor, and axial muscles in the cat. J. Comp. Neurol. 382, 46–76. 10.1002/(sici)1096-9861(19970526)382:1<46::aid-cne4>3.0.co;2-k 9136811

[B117] Vinay L. Brocard F. Clarac F. Norreel J. C. Pearlstein E. Pflieger J. F. (2002). Development of posture and locomotion: an interplay of endogenously generated activities and neurotrophic actions by descending pathways. Brain Res. Brain Res. Rev. 40, 118–129. 10.1016/s0165-0173(02)00195-9 12589911

[B118] Wagner F. B. Mignardot J.-B. Le Goff-Mignardot C. G. Demesmaeker R. Komi S. Capogrosso M. (2018). Targeted neurotechnology restores walking in humans with spinal cord injury. Nature 563, 65–71. 10.1038/s41586-018-0649-2 30382197

[B119] Wan Y. Wei Z. Looger L. L. Koyama M. Druckmann S. Keller P. J. (2019). Single-cell reconstruction of emerging population activity in an entire developing circuit. Cell 179, 355–372. 10.1016/j.cell.2019.08.039 31564455 PMC7055533

[B120] Ward S. R. Eng C. M. Smallwood L. H. Lieber R. L. (2009). Are current measurements of lower extremity muscle architecture accurate? Clin. Orthop. Relat. Res. 467, 1074–1082. 10.1007/s11999-008-0594-8 18972175 PMC2650051

[B121] Warp E. Agarwal G. Wyart C. Friedmann D. Oldfield C. S. Conner A. (2012). Emergence of patterned activity in the developing zebrafish spinal cord. Curr. Biol. 22, 93–102. 10.1016/j.cub.2011.12.002 22197243 PMC3267884

[B122] Wenger N. Moraud E. M. Gandar J. Musienko P. Capogrosso M. Baud L. (2016). Spatiotemporal neuromodulation therapies engaging muscle synergies improve motor control after spinal cord injury. Nat. Med. 22, 138–145. 10.1038/nm.4025 26779815 PMC5061079

[B123] Wenger N. Moraud E. M. Raspopovic S. Bonizzato M. DiGiovanna J. Musienko P. (2014). Closed-loop neuromodulation of spinal sensorimotor circuits controls refined locomotion after complete spinal cord injury. Sci. Transl. Med. 6, 255ra133. 10.1126/scitranslmed.3008325 25253676

[B124] Yakovenko S. Mushahwar V. VanderHorst V. Holstege G. Prochazka A. (2002). Spatiotemporal activation of lumbosacral motoneurons in the locomotor step cycle. J. Neurophysiol. 87, 1542–1553. 10.1152/jn.00479.2001 11877525

[B125] Yokoyama H. Hagio K. Ogawa T. Nakazawa K. (2017). Motor module activation sequence and topography in the spinal cord during air-stepping in human: insights into the traveling wave in spinal locomotor circuits. Physiol. Rep. 5, e13504. 10.14814/phy2.13504 29180480 PMC5704080

[B126] Yue G. H. Ranganathan V. K. Siemionow V. Liu J. Z. Sahgal V. (1999). Older adults exhibit a reduced ability to fully activate their biceps brachii muscle. J. Gerontol. A Biol. Sci. Med. Sci. 54, M249–M253. 10.1093/gerona/54.5.m249 10362008

[B127] Zhvansky D. S. Sylos-Labini F. Dewolf A. Cappellini G. d’Avella A. Lacquaniti F. (2022). Evaluation of spatiotemporal patterns of the spinal muscle coordination output during walking in the exoskeleton. Sensors (Basel) 22, 5708. 10.3390/s22155708 35957264 PMC9370895

